# Questionnaire on adaptation to type 1 diabetes among children and its relationship to psychological disorders*

**DOI:** 10.1590/1518-8345.2759.3088

**Published:** 2018-11-14

**Authors:** Laura Lacomba-Trejo, Selene Valero-Moreno, Sara Casaña-Granell, Vicente Javier Prado-Gascó, Marián Pérez-Marín, Inmaculada Montoya-Castilla

**Affiliations:** 1 Universitat de València, Facultad de Psicología, València, Comunidad Valenciana, Spain.

**Keywords:** Adaptive Response, Diabetes Mellitus Type I, Pediatrics, Anxiety, Depression, Psychometric

## Abstract

**Objective:**

to study the psychometric properties of an adaptive disease response questionnaire for use with Spanish children with type 1 diabetes; to analyse this response in this sample and to observe the relationship between adaptive response and levels of anxiety-depression.

**Method:**

a total of 100 patients with type 1 diabetes aged between nine and 16 years (M=12.28, SD=1.78) participated in the study, of which 59% were children. Data was collected in public hospitals via interviews using the Adaptive Disease Response Questionnaire and Anxiety and Depression Scale. The data was analysed using Pearson correlations, multiple hierarchical linear regressions, Student’s t Test for independent samples, and Cohen’s d effect size to determine reliability and validity.

**Result:**

the instrument was shown to have adequate psychometric properties. Adaptive response was generally high. Adaptive response is negatively related to emotional distress, being a better predictor of depression than of anxiety. There was no association betwee adaptation and sex and age.

**Conclusion:**

promoting a better adaptive response appears to reduce emotional distress, especially in the case of depression, regardless of the age or gender of the patients.

## Introduction

Type 1 diabetes type 1 diabetesis the most common endocrine disease in children and adolescents^1^ ^-^ ^3^. It is characterized by a deficit in insulin production and requires daily insulin injections to control glucose levels^4^. For this reason, the literature on type 1 diabetes has tended to focus on evaluating medical aspects of disease control^1^, such as levels of glycosylated haemoglobin (HbA1c) as a biological marker of glycaemic control over the last 3 months^5^ and self-monitoring of blood glucose levels^6^, to the detriment of psychological aspects, which exert a decisive influence on patient/family well-being and quality of life, notably the so-called adaptive response to the disease^7^.

The adaptive response to chronic diabetes conditions is defined as the *“degree of psychosocial adjustment of the patient’s behaviour, emotional state and assessment in relation to his or her condition* ^”(^ ^8^. Tackling chronic disease in children is a complex and multidimensional process that begins when the patient is diagnosed and must cope with the changes and/or emerging complications in all dimensions of life and adjust to his/her condition in order to have the best possible quality of life^6^.

Adjustment to type 1 diabetes involves not only mastering the tasks the patient must undertake to adapt to the disease, such as glycaemic control, healthy-eating, insulin administration, and practising regular exercise^6^, but also maintaining adequate functional and emotional health status, preventing psychological disorders such as anxiety or depression and low negative affect, and maintaining a positive perception of quality of life^9^.

In this sense, psychological factors have been proved to play an important role in disease adaptation among children with type 1 diabetes^10^. In this respect, it is common for type 1 diabetes to have emotional impacts, notably depression and anxiety disorders ^11^ ^-^ ^12^, particularly among adolescents^13^.

Psychological adjustment is intimately linked to adherence to treatment, prognosis, and the physical and mental health of patients and their families^14^. In this sense, a poor emotional adjustment is associated with poor metabolic control^5^ ^,^ ^14^ ^-^ ^16^, causing an increase in medical complications and barriers to treatment adherence, ultimately leading to a deterioration in the patient’s condition^17^. In this respect, the American Academy of Paediatrics (AAP) maintains that it is necessary to address both the medical demands and the psychological needs of paediatric patients in order to improve adaptation to chronic disease^18^ ^-^ ^19^.

Psychological adjustment is a dynamic and continuous process in which the patient’s mental health status changes as treatment demands, life threat level, disability, and prognosis change^9^.

Likewise, studies have shown that sociodemographic variables such as age and sex may influence adjustment^11^ ^,^ ^20^. In this sense, emotional impacts seem to be more pronounced among girls^20^ ^-^ ^21^; however, the association between age and emotional impacts during childhood and adolescence is less clear^22^ ^-^ ^23^. In this respect, studies^22^ indicate that risk is greater between the ages of 10 and 13 years, while others ^11^ ^,^ ^23^ suggest that impacts increase with age.

Despite the high prevalence of type 1 diabetes in among children^2^ and its serious consequences^19^, efforts geared towards assessing adaptive response among Spanish patients have tended to focus on adults or elderly type 2 diabetes patients^6^.

Another research challenge when it comes to type 1 diabetes in children is the small range of instruments for measuring adaptive response^8^. Existing instruments focus mainly on aspects related to quality of life or emotional symptoms^24^, with the use of generic instruments across all types of chronic diseases^9^. This problem is exacerbated in the Spanish context, where instruments developed in the English language^24^ ^-^ ^25^ tend to be used, which, in general, fail to give due consideration to the psychological aspects of the adaptive response to type 1 diabetes.

One of the few instruments specifically designed for type 1 diabetes translated into Spanish is the Adaptive Response Questionnaire for Diabetic Patients (ARD)^8^. This instrument evaluates patients perceptions of the severity of their illness, the factors that hinder treatment adherence, the extent to which patient behaviour favours treatment adherence, discomfort associated with the illness, and its psychological consequences^8^. However, despite being the most widely used instrument in this context, there is a lack of research in the following areas: the psychometric properties of the instrument; its effectiveness in analysing the common emotional impacts of the disease (anxiety and depression); the association between sex and age and emotional impacts. In light of the above, this study is especially relevant given the scarce literature on adaptive response in children with type 1 diabetes and, above all, due to the need for tools tailored to the Spanish context that can be used by a variety of different health professionals.

This study therefore aimed to examine the psychometric properties of the Adaptive Disease Response Questionnaire applied with a sample of Spanish children with type 1 diabetes, analyse the adaptive response of these patients, and investigate the relationship between the adaptive response and levels of anxiety and depression. The results constitute an important input for the development and enhancement of interventions to promote quality nursing care and improve the health of patients and their families.

## Method

The sampled comprised 113 children diagnosed with type 1 diabetes referred to the paediatric endocrinology units of three reference hospitals in the Valencian Community, Spain. After a review of the medical records of the interviewed patients and consulting healthcare staff, 13 patients were excluded because they did not meet the following inclusion criteria: children diagnosed within the last six months; no other previous physical or psychological illness that could interfere with their adaptation to diabetes.

The study was approved the Ethics Committee of the University of Valencia (H143497959393931) and by the bioethics committees of the participating hospitals. The study objectives and procedures were explained to the participants and their parents/guardians, who signed an informed consent form guaranteeing confidentiality and freedom to withdraw from the study at any time.

The information was collected via interviews employing an instrument composed of an *ad hoc* register and two instruments standardized by the same professional in all cases. The variables analysed and instruments used were:

*Ad hoc* recording: patient age and sex; the existence of a secondary diagnosis; length of time since diagnosis and in treatment; number of disease-related hospitalizations.

Adaptive response to the disease diabetes patients: Adaptive response was evaluated using the Adaptive Response Questionnaire to the Disease in Diabetes Patients^8^. This 32-item instrument was developed and applied to Cuban adults to assess the factors influencing psychological and social responses to the disease among diabetes patients, taking into account cognitive, emotional and behavioural aspects. This quick and easy-to-use questionnaire enables an adjusted assessment of adaptive response among diabetes patients and can thus be used by health professionals who have most contact with patients and their families such as nurses. However, it has yet to be adapted or validated for use with Spanish patients or children. The questionnaire consists of the following subscales:

- Assessment of disease severity (items 1, 2, 3, 4, 5, 7 and 9), which refers to the degree to which the patient perceives the disease as being detrimental in terms of quality and duration of life and potentially dangerous in terms of its consequences.
- Barriers to treatment adherence (items 6, 10, 12, 13 and 31) or aspects of treatment that the patient considers negative or difficult to adhere to.
- Health behaviour (items 8, 11, 24, 25 and 26), which assesses the degree to which the patient’s behaviour is favourable to treatment adherence.
- Discomfort associated with the disease (items 16, 27 and 28), or frequency of onset and intensity of physical symptoms of type 1 diabetes.
- Psychological repercussions (items 14, 15, 17, 18, 19, 20, 21, 22, 23, 29 and 30), referring to the degree to which the patient’s self-esteem is affected by feelings of worthlessness and shame related to the illness.

The original instrument consists of 32 items organised into five dimensions. However, based on expert judgment it was item 25 was removed from the questionnaire because it did not fit the reality of the Spanish population, resulting in the 31-item version used in this study. Each item has a range of response options scored from 0 to 6. The overall score for the questionnaire is calculated based on the sum of the scores obtained for each item and ranges from 0 to 76 on the original scale and 0 to 75 in the version used for this study. The lower the score the greater disease severity, problems associated with the disease, and psychological impact and poorer health behaviour. According to the authors, a score of over 64.6 points is considered a good adaptive response. Given the exclusion of one of the items, for the purposes of this study the cut-off point was set at 63.5. The original authors did not report the psychometric properties of the instrument.

Emotional symptoms in patients diagnosed with a disease: This aspect was evaluated using the Hospital Anxiety and Depression Scale (HADS)^26^, a 14-item screening tool using a three-point Likert-type scale, where 0 is the minimum score and 3 the maximum. The tool detects anxious (odd numbered items) and depressive (even numbered items) symptoms felt over the week prior to the interview. The sum of the two scales represents the overall emotional distress score. In general, the higher the score the greater emotional involvement and the higher levels of anxiety and depression. Previous studies that used this instrument on the Spanish population showed that it had adequate psychometric properties^27^ with reliability coefficients of between 0.68 and 0.93 (Mα= 0.83) for anxiety and 0.67 and 0.90 (Mα= 0.82) for depression. Reliability was also shown to be good when used with children and adolescents, although the coefficient was somewhat lower for depression^28^ ^-^ ^29^. The alpha values of the version used in the present study ranged from 0.58 to 0.77.

To analyse the data from the adaptive response questionnaire we elaborated scales based on the percentiles for the total population and according to sex and age. The relationships between disease adaptation and emotional distress were then analysed using Pearson correlations and multiple hierarchical linear regressions. Finally, to analyse the effect of sociodemographic variables, mean differences were calculated using Student’s t Test for independent samples and Cohen’s d effect size, Pearson’s correlations, and multiple hierarchical linear regressions by sex and age groups (preadolescents: 9-12 years; adolescents: 12-16 years). These analyses were performed using SPSS® Statistics version 24.0.

## Results

With respect to age, 59% of the sample were children aged between 9 and 16 years (M=12.28, SD=1.78), while 53% were pre-adolescents (9-12 years) and 47% adolescents (12-16 years). All patients had been diagnosed with type 1 diabetes within the last six months; more specifically the length of time since diagnosis ranged from 6 to 95 months (M=71.38; SD=104.66). Secondary diagnoses were found among 15.9% of the sample, namely: allergy, celiac disease, hypothyroidism, migraines or headaches, juvenile idiopathic arthritis, retinite pigmentosa, and reflux nephropathy. Time in treatment varied between 0 and 959 months (Range 0-959), with an average time of 70.70 months (SD=104.97), 5 years. The number of diabetes-related hospital admissions ranged between 0 and 40 admissions (Range 0-40), with an average of 2.18 (SD=5.10).

With regard to emotional distress (anxiety and depression), the overall mean for emotional discomfort and the subscales anxiety and depression were low (M= 6.79, SD= 4.84; M= 5.22, SD= 3.46; and M= 1.57, SD= 1.91, respectively). However, based on HADS interpretive criteria, 23% of the patients presented symptoms of anxiety and 8% had an anxiety disorder, while 1% of patients suffered from clinical depression and 2% had an emotional distress disorder.

To determine the psychometric properties of the adaptive response questionnaire, several exploratory factorial analyses (EFA) were conducted using the principal component method. The best solution was a single factor explaining 24.26% of the variance. The reliability analyses based on the five original dimensions proposed by the authors resulted in low reliability (α<0.70). Based on the results of the EFA we therefore went on to analyse reliability using a single global dimension, which we have called *general adaptation to the type 1 diabetes*, obtaining an acceptable coefficient (α=0.77). The results of the analysis of the elements suggested that no items needed to be removed to improve the reliability of the tool.

We then proceeded to analyze adaptation to the disease by the patients. Based on the cut-off point mentioned above, the adaptive response given by the patients was coded, with scores above 64.6 indicating an adaptive response. The results showed that 24.5% of the patients demonstrated poor adaptation to the disease. Table 1 shows the average scores obtained in each of the subscales of the adaptive response questionnaire.

**Table 1:** Descriptive statistics of ARD* factors in children with type 1 diabetes Valencia, Spain, 2016-2017

	M^†^	SD^§^	Min^||^	Max^¶^
F1. Assessment of severity	12.52	3.37	3	19
F2. Barriers to adherence	12.18	3.23	2	17
F3. Health behaviour	10.87	1.27	7	13
F4. Discomfort associated with the disease	3.90	1.03	1	5
F5. Psychological impact	17.83	3.44	7	24
F6. Total adaptation	57.30	8.96	29	73

*ARD - adaptive response to disease; †M - mean; §SD - standard deviation; ||Min. - minimum; ¶Max. - maximum

Subsequently, in order to facilitate the interpretation of the data by health professionals, scales were developed for adaptation to the disease, according to sex and age, as indicated in Table 2.

**Table 2:** Scales of the adaptive response to the disease according to age and sex in children with type 1 diabetes. Valencia, Spain, 2016-2017

Pre-adolescence (9-12 years) n^†^=53			Percentile	Adolescence (12-16 years) n^†^=47		
Total	Children (n^†^=51)	Girls (n^†^=47)		Total	Children (n^†^=30)	Girls (n^†^=17)
70	69.2	71.5	90	66.2	66.9	65.8
66.6	67	66	80	64	64.8	62.2
65	66	61	70	61	61	61
62	64.6	59	60	59.8	60.6	58.8
59	62	58	50	58	58	55
57	58.2	56	40	55	55.8	53.2
52	54.8	51	30	53	53.3	51.8
51	51	50	20	51	51.2	43.6
47.4	49	40.5	10	43	45	40.8

†n - group sample size

After analysing the response to the disease, we used Pearson correlation coefficients and three multiple hierarchical regressions to analyze the relationship between patient adaptation and emotional distress (anxiety and depression) measured using the HADS (Table 3).

**Table 3:** ARD* correlations using HADS^†^ in children with type 1 diabetes, Valencia, Spain, 2016-2017

Correlations	HADS^†^			Age
	A^§^	D^||^	ED^¶^	
ARD*(n**=100)	-0.55^††^	-0.70^††^	-0.67^††^	-0.03
ARD*_boys_(n**=59)	-0.44^††^	-0.78^††^	-0.68^††^	-0.06
ARD*_girls_ (n**=41)	-0.63^††^	-0.62^††^	-0.66^††^	-0.18

*ARD - Adaptive response to disease; †HADS - Hospital Scale of Anxiety and Depression; §A - anxiety; ||D - depression; ¶ED - emotional distress; **n - group sample size; ††p - significance level p≤0.01

Pearson’s correlations showed that adaptation showed a negative, significant (*p*≤.01) and moderate or strong association with anxiety, depression and emotional distress.

Three hierarchical multiple regression analyses were then performed using age and overall ARD adaptation as predictor variables and the domains of anxiety, depression and emotional distress (HADS) as criterion variables. In all cases the first step included age, and in the second the overall ARD score, the main results of the final models were as follows:

a) With respect to prediction of anxiety, the inclusion of age did not improve the model (ΔR^2^=0.00, *p*=0.92). With the inclusion of the overall ARD score, the model improved significantly (ΔR^2^=0.20, *p*≤.001). More specifically, after the final step, anxiety intensity was directly predicted negatively using only the overall ARD score *(*β = -0.45, *p* ≤.001).
b) With regards to depression, the inclusion of age did not significantly increase the variance of depression (ΔR^2^=0.01, *p=0*.34). After adding the overall ARD score, the model improved significantly (ΔR^2^=0.40, p≤.001). More specifically, after the final step, depression was directly predicted negatively using only the overall ARD score (β =-0.63, p≤.001).
c) In terms of emotional distress, the inclusion of age did not significantly improve prediction (ΔR^2^=0.01, *p=*0.65). With the inclusion of the overall ARD score, the model improved significantly (ΔR^2^=0.33, p≤.001). Emotional distress was directly predicted negatively using only the overall ARD score (β =-0.57, *p* ≤.001).

After analysing the impact of adaptation on emotional distress, we analysed the impact of age and sex on these relationships. To this end, we analysed the association between adaptation and sex and age group. The Pearson correlations were then analysed by age and sex, and multiple hierarchical regressions were performed considering age, sex and the overall ARD score.

**Figure 1:**
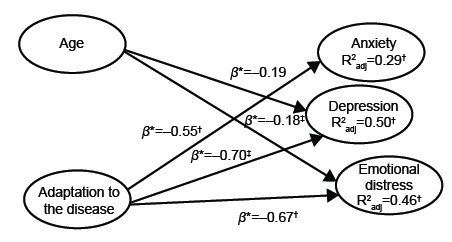
Relationship model, Valencia, Spain, 2016-2017

The results show that there was no association between adaptation and sex (t_96_= 1.38; *p=0*.17, M_Boys_=58.35, SD_Boys_=8.43, M_Girls_=55.83, SD_Girls_=9.56, *d=0*.30*)* and age (t_96_=0.83; *p=0*.41, M_early adolescence_=58.02_,_ SD_early adolescence_ =9.75, M_adolescence=_56.51, SD_adolescence=_=8.04, *d=0*.17*)*. Likewise, there was a statistically significant (p≤.01) negative correlation between anxiety, depression and emotional distress and adaptation among both boys and girls, with higher correlation coefficients among boys for depression and emotional distress. For the overall group, depression showed the highest correlations (r=-0.78, p≤.001), followed by emotional distress (r=-0.68, p≤.001), and anxiety (r=-0.44, *p=0*.01). Similar correlation coefficients were observed for the three dimensions tested by HADS and overall patient adaptation among girls: emotional distress (r=-0.66, p≤.001), anxiety (r=-0.63, p≤.001), and depression (r=-0.62, p≤.001) (Table 3). Finally, with respect to the hierarchical multiple linear regression models, sex was included in the first step, age in the second, and overall ARD score in the third. In the first step sex resulted in a non-significant prediction of 2% of anxiety variance (F=1.07, *p=0*.30), 2% of depression (F=1.74, *p=0*.19), and 2% of emotional distress (F=1.62, *p=0*.27). The inclusion of age in the second step as a predictor variable did not significantly improve the explanation of the model as the increase in R^2^adj ranged from 0.02 to 0.03 (*p*≥.05*)* in all the dimensions tested by HADS. However, age showed a statistically significant negative beta coefficient for depression (β=-0.19; *p*≤0.05) and emotional distress (β=-0.18; *p*≤0.05) in this step. Finally, the inclusion of adaptation in the third step as a predictor variable significantly improved the explanation of the model: by 29% for anxiety (F=10.65, β=-0.55, p≤.001); 47% for depression (F=24.97, β=-0.70, p≤.001); and 44% for emotional distress (F=21.19, β=-0.67, p≤.001). Therefore, the only predictor of emotional distress is the overall ARD score, which represents the level of general adaptation of diabetes patients and is a negative predictor of emotional distress (Figure 1).

*β - standardized beta coefficient; † significance level p≤.01; ‡ significance level p≤.05

## Discussion

With respect to the reliability of the Adaptive Response Questionnaire to the Disease in Diabetes Patients, the results of the analysis suggest that the instrument shows adequate level of internal consistency for use on the Spanish population. With regard to the psychometric properties of the instrument, the findings show that, unlike the original structure proposed by the authors^8^, the instrument made up of 31 items grouped in a single dimension is reliable and valid. It is important to note that this instrument had not been validated for use on the Spanish population up till now, meaning that this study is of particular relevance.

With regard to the emotional impact of type 1 diabetes on patients, it was observed that 24.5% have difficulties in adapting to the disease, which can cause low treatment adherence and emotional problems related to the adjustment to the disease^10^ ^-^ ^11^ ^).^ These results are in line with studies that show that poor adjustment rates cause an increase in medical complications and create barriers to treatment adherence ^5^ ^,^ ^14^ ^-^ ^16^, which ultimately leads to a deterioration in the patient’s condition^17^.

Given the dynamic nature and complexity of the adaptive response to type 1 diabetes throughout the course of the disease^9^, scales were drawn up for sex and age categories (pre-adolescence and adolescence) to help health professionals interpret the scores, thus enhancing care and improving treatment adherence and, ultimately, the health of patients and their families.

The next step was to analyse the relationship between the adaptive response to the illness and emotional distress. As expected, and according to the existing literature^11^ ^-^ ^13^ ^),^ the results show that adaptation to the disease is negatively and significantly related to emotional distress, that is to say adaptation to diabetes has an inverse influence on symptoms of anxiety and depression, especially in the case of depression, where adaptation to the disease has greater predictive power. In this sense, the better the adaptation to diabetes the less likely the patient is to experience negative emotional symptoms. This is a positive factor because anxiety and depression at these ages is a risk factor for the proper metabolic control of the disease^16^, increasing the likelihood of medical complications^5^ ^,^ ^14^ ^-^ ^15^. This in turn has a negative impact on the physical and mental health and quality of life of patients and their families.

Finally, the results show that there is no association between sex and age and adaptation to the disease or emotional distress, which is partially in line with previous studies ^20^ ^-^ ^21^. In this respect, the girls in this study were not shown to demonstrate poorer psychological adjustment, as in other studies. However, there was no association between age and adjustment, as indicated by previous research^22^ ^-^ ^23^. Likewise, both boys and girls showed high positive correlations across the variables, particularly the boys. Finally, neither sex nor age were predictors of emotional distress, which corraborates previous studies^21^ ^-^ ^23^.

The findings of this study serve to fill a gap in the literature on validated instruments for use with Spanish children. This instrument comprises a quick and easy-to-use tool for assessing the adaptive response of patient to type 1 diabetes.

## Strengths and limitations

The main strength of this study is the validation of a reliable instrument encompassing psychological adjustment for use with Spanish type 1 diabetes patients, which also takes into account variables such as sex and age. However, the study is not without its limitations, one of which is the sample size and sampling procedure, which are not probabilistic, making it difficult to generate the data. However, the sample size used by this study is similar and in some cases larger than those of other studies with this population group, which is probably due to the difficulties experiences in accessing this group. Further more in-depth research should be undertaken with larger samples. Another of the limitations lies in the fact that data collection was limited to questionnaires. Future research should consider the use of objective medical indicators (for example Hba1c and cortisol levels) and their relationship with patient adaptation. In spite of the above, the present study provides valuable input given the lack of instruments adapted and validated for use with the Spanish population.

## Conclusions

The results of this study provided a better understanding of the current state of research on type 1 diabetes in this age group.

The findings show that sociodemographic variables do not influence adaptive response to type 1 diabetes in children and that adequate adaptive response is negatively related to the presence of psychological disorders (anxiety and depression). Thanks to this study, nursing professionals and other related areas will now have a reliable and valid instrument to assess adaptive responses to type 1 diabetes among children.

Addressing these aspects allows health professionals, particularly nurses, to assess emotional adaptation to diabetes in children with the ultimate aim of ensuring they have the best possible quality of life.

This study provides important inputs for the future development of nursing research, both for readers and practitioners, by suggesting gaps in the literature that could in turn guide current trends and future directions in research.

As international organizations such as the World Health Organization and the American Psychiatric Association suggest, there is a need to focus on adjustment to chronic disease in children, not only from a medical point of view but also multidimensionally, reflecting the need to build valid instruments for use with this population group. This study is therefore particularly relevant because it was able to validate an adaptive response questionnaire for use with the Spanish population, which is something that had not been carried out until now, thus providing a useful and easy-to-use tool that shows that poor adaptive response is related to the presence of emotional symptoms and that adaptive response is similar in patients with type 1 diabetes regardless of the patient’s sex or age. In light of the above, further research is needed together with initiatives to address this problem and the promotion of training programmes to equip health professionals with the necessary skills and knowledge to enhance the adaptation of patients to type 1 diabetes and deliver high quality nursing care.
